# MicroRNA-802 induces hepatitis B virus replication and replication through regulating SMARCE1 expression in hepatocellular carcinoma

**DOI:** 10.1038/s41419-019-1999-x

**Published:** 2019-10-14

**Authors:** Yuanyuan Wang, Jingmei Cao, Shiyun Zhang, Lei Sun, Yi Nan, Hong Yao, Jian Fan, Li Ying Zhu, Lei Yu

**Affiliations:** 1 Department of Infectious Disease, The Fourth Hospital of Harbin Medical University, Harbin, 150001 Heilongjiang China; 2 Department of Gastroenterology, Zibo Central Hospital, Zibo, 255000 Shandong China; 3 Department of General Surgery, Linyi People’s Hospital, Linyi, 276000 Shandong China; 4 Department of Ophthalmology, The Fourth Hospital of Harbin Medical University, Harbin, 150001 Heilongjiang China; 5 Traditional Chinese Medicine College of Ningxia Medical University, Yinchuan, 750004 China; 6 Division of Infectious Diseases, Brigham and Women’s Hospital, Boston, Massachusetts & Harvard Medical School, Boston, Massachusetts USA

**Keywords:** Cancer, Cell biology

## Abstract

Growing evidences have indicated that microRNAs (miRNAs) can regulate hepatitis B virus (HBV) expression and replication, playing crucial roles in the development of HBV infection. Until now, the functional role and mechanism of miR-802 in HBV replication and expression remain unknown. We indicated that miR-802 expression was upregulated in the HBV-associated hepatocellular carcinoma (HCC) tissues compared with the adjacent noncancerous samples. In addition, we showed that the SMARCE1 expression level was downregulated in the HBV-associated HCC tissues compared with the adjacent noncancerous samples. miR-802 expression was negatively related with MARCE1 expression in HBV-associated HCC tissues. Moreover, miR-802 expression was upregulated, and SMARCE1 expression was downregulated in the HBV-infected HepG2.2.15 cells. Ectopic expression of miR-802 significantly enhanced HBV DNA replication, while knockdown of miR-802 significantly decreased HBV DNA replication. We showed that overexpression of miR-802 promoted HbsAg and HbeAg expression, while inhibition of miR-802 decreased HbsAg and HbeAg expression. Furthermore, we indicated that ectopic expression of SMARCE1 suppressed HBV DNA replication and decreased the expression level of HbsAg and HbeAg. Finally, we showed that overexpression of miR-802 promoted HBV DNA replication through regulating SMARCE1 expression. These results suggested the important roles of miR-802 on HBV expression and replication, which may shed new light on the development of treatment for HBV.

## Introduction

Hepatitis B virus (HBV) is a member of the Hepadnavirus family and its infection is a big public health concern^1–3^. HBV infection is the first risk factor of hepatocellular carcinoma (HCC) according to previous epidemiological data^4–9^. Until now, HBV infection remains highly prevalent worldwide, with about 240 million infected cases^10,11^. Utilization of nucleoside analogs and a-interferon suppresses HBV replication to some degree with significant adverse effects and high cost^12–14^. Thus, it is crucial to study the mechanism of durable virus infection to develop new antiviral strategies.

MicroRNAs (miRNAs) are an abundant group of noncoding, short RNAs that regulate gene expression through mRNA degradation or translational repression^15–18^. miRNAs regulate their target gene expression via binding to the 3′UTR (3′ untranslated regions)^19–21^. It was proved that miRNAs play important roles in several cell biologies such as cell angiogenesis, apoptosis, proliferation, invasion, and immunity in cancer^22–24^. Previous studies indicated that miRNAs were deregulated in cancer and functioned as oncogenes or tumor suppressors^25–27^. A lot of miRNAs, such as miR-101, miR-125a-5p, miR-501, and miR-141, were shown to be involved in the modulation of HBV replication^28–30^. However, the relationship between miR-802 expression and HBV infection and replication is still unknown.

In our study, we demonstrated that the expression of miR-802 was upregulated in the HBV-associated HCC tissues compared with the adjacent noncancerous samples. In addition, we showed that the SMARCE1 expression level was downregulated in the HBV-associated HCC tissues compared with the adjacent noncancerous samples. Moreover, miR-802 expression was upregulated, and SMARCE1 expression was downregulated in the HBV-infected HepG2.2.15 cells. Ectopic expression of miR-802 significantly enhanced HBV DNA replication, while knockdown of miR-802 significantly decreased HBV DNA replication.

## Materials and methods

### Clinical samples

Thirty human HBV-associated HCC tissues and adjacent noncancerous samples were obtained from The Fourth Hospital of Harbin Medical University (Harbin, China). These tissues were immediately snap-frozen in liquid nitrogen and stored until used. Our study was approved by The Fourth Hospital of Harbin Medical University Ethics Committee, and written informed consent was collected from all subjects.

### Cell culture and transfection

Human HCC cell line HepG2.2.15 with the HBV infection and HCC cell line HepG2 without HBV infection were collected from ATCC (American Type Culture Collection, Manassas, USA). The cell was kept in DMEM (Dulbecco’s modified Eagle’s medium, Gibco, NY, USA) supplemented with FBS (fetal bovine serum, 10%, Gibco, NY, USA) and streptomycin/penicillin (Invitrogen, CA, USA). Anti-miR-control, anti-miR-802, miR-802 mimic, scramble, pcDNA-SMARCE1, and pcDNA-control were transfected into these cells by using Lipofectamine 3000 following the manufacturer’s protocol.

### RNA isolation and quantitative real-time PCR (qRT-PCR)

Total RNAs from HepG2.2.15, HepG2 cell, or samples were isolated by using TRIzol Reagent (Invitrogen, NY, USA) following the manufacturer’s instruction. The expression level of miR-802 and SMARCE1 was analyzed with SYBR Green Kit (Takara, Dalian, China) on the ABI 7000 (Applied Biosystems, Foster City, CA, USA) according to the manufacturer’s instruction. GAPDH and U6 acted as the internal controls for mRNA and miRNA, respectively.

### Luciferase reporter gene assay

The SMARCE1 3′UTR containing predicted mutant or wild-type miR-802-binding site was amplified with PCR from genomic DNA of HepG2.2.15 cell and cloned into luciferase reporter plasmid pGL3 (Promega, WI, USA) to establish pGL3-WT-SMARCE1 or pGL3-MUT-SMARCE1. This luciferase reporter was respectively transfected into HepG2.2.15 cell by using Lipofectamine 3000 (Invitrogen, USA) along with phRL-TK vector (Promega), pGL3-WT-SMARCE1 or pGL3-MUT-SMARCE1, and miR-802 mimic or scramble. Luciferase activity was detected with the Dual-Luciferase Reporter analysis System (Promega) after 48 h post transfection.

### Western blot analysis

Total protein from the HepG2.2.15 cell was obtained by using RIPA reagents (Thermo Scientific, IL, USA). The concentration of protein was determined with the BCA protein kit (Thermo Scientific, Rockford, USA). An equal amount of protein was managed by SDS-PAGE and transferred to PVDF (polyvinylidene fluoride) membrane. After blocking in TBST containing 5% milk, the membrane was incubated with primary antibodies (anti-SMARCE1 and anti-GAPDH, dilution, 1:1000; Abcam, MA, USA) overnight at 4 °C. After incubation with secondary antibodies, the signal of blot was analyzed by the enhanced chemiluminescence detection kit (Ecl, Healthcare Biosciences, Pittsburgh, PA). GAPDH was used as the loading control.

### HBV replication and gene expression analysis

HBV DNA in the HCC cell culture supernatants was separated with the Column Viral DNAout kit (TIANDZ, China) according to the manufacturer’s instruction and determined with real-time PCR assay as described previously. The expression levels of hepatitis B e antigen (HBeAg) and hepatitis B surface antigen (HBsAg) in HCC cell supernatants were detected by using ELISA (enzyme-linked immunosorbent assay) kit (Kehua Biotech, Shanghai, China).

### Statistical analysis

All the results were determined by using SPSS 18.0 software (SPSS, Chicago, USA). The data value was expressed as mean ± standard deviation (SD). Comparison of significant difference between two groups was utilized by Student’s *t* test. *P* value < 0.05 was represented as statistically significant.

## Results

### The expression level of miR-802 was upregulated in HBV-associated HCC tissues

**Fig. 1:**
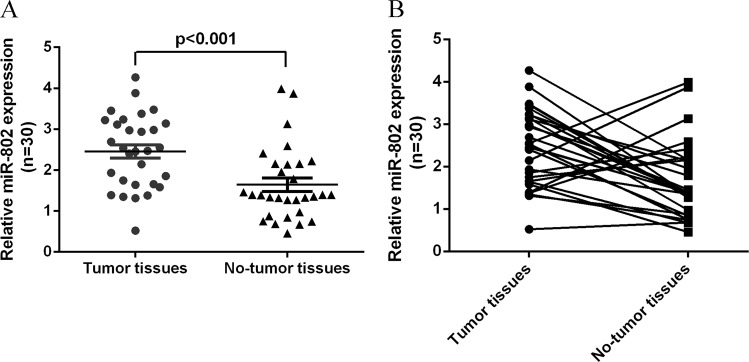
The expression level of miR-802 was upregulated in HBV-associated HCC tissues. **a** The expression of miR-802 in HBV-associated HCC and adjacent noncancerous samples was determined by qRT-PCR assay. U6 was used as the internal control. **b** miR-802 was upregulated in 22 patients (22/30; 73.3%) compared with adjacent noncancerous tissues

First, we measured the expression level of miR-802 in HBV-associated HCC tissues and the adjacent noncancerous samples. We showed that miR-802 expression was upregulated in the HBV-associated HCC tissues compared with the adjacent noncancerous samples by using qRT-PCR analysis (Fig. 1a). In addition, we indicated that miR-802 was upregulated in 22 patients (22/30; 73.3%) compared with adjacent noncancerous tissues (Fig. 1b). The expression level of miR-802 was not correlated with the levels of serum HBsAg and HbeAg.

### The expression level of SMARCE1 was downregulated in HBV-associated HCC tissues

**Fig. 2:**
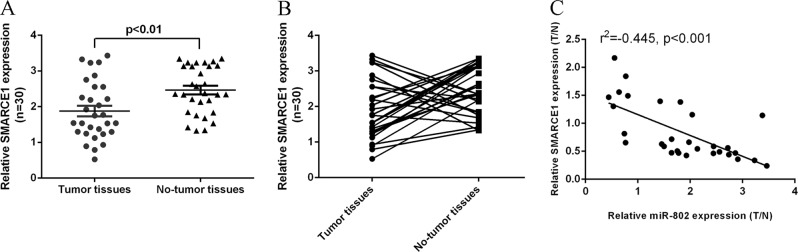
The expression level of SMARCE1 was downregulated in HBV-associated HCC tissues. **a** The expression of SMARCE1 was measured in the HBV-associated HCC tissues and adjacent noncancerous samples by using qRT-PCR analysis. **b** SMARCE1 expression was upregulated in 20 patients (20/30; 6.67%) compared with the adjacent noncancerous tissues. **c** The expression of miR-802 was negatively related with the expression of SMARCE1 in HBV-associated HCC tissues

Second, we determined the expression level of SMARCE1 in HBV-associated HCC samples and the adjacent noncancerous samples. We indicated that SMARCE1 expression level was downregulated in the HBV-associated HCC tissues compared with the adjacent noncancerous samples by using qRT-PCR analysis (Fig. 2a). Moreover, we indicated that SMARCE1 expression was upregulated in 20 patients (20/30; 6.67%) compared with the adjacent noncancerous tissues (Fig. 2b). The expression level of SMARCE1 was not correlated with the levels of serum HBsAg and HbeAg. In addition, we showed that miR-802 expression was negatively related with the expression of SMARCE1 in HBV-associated HCC tissues (Fig. 2c).

### miR-802 expression was upregulated while SMARCE1 expression was downregulated in the HBV-infected cells

**Fig. 3:**
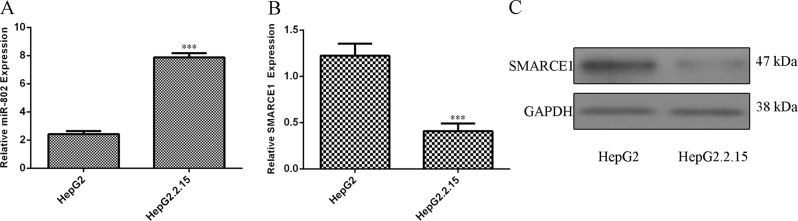
miR-802 expression was upregulated while SMARCE1 expression was downregulated in the HBV-infected cells. **a** The expression of miR-802 in the HBV-infected HepG2.2.15 cell and HepG2 cell was determined by qRT-PCR analysis. **b** The expression level of SMARCE1 in the HBV-infected HepG2.2.15 cell and HepG2 cell was measured by qRT-PCR assay. **c** The protein expression of SMARCE1 was measured by western blot. GAPDH was used as the internal control. ***p < 0.001

The expression levels of miR-802 in HepG2 cells and HBV-infected HepG2.2.15 cell were measured by qRT-PCR analysis. We showed that the expression of miR-802 was upregulated in the HepG2.2.15 cell compared with the HepG2 cell (Fig. 3a). The expression level of SMARCE1 was downregulated in HepG2.2.15 cell compared with the HepG2 cell (Fig. 3b). We also found that the protein expression of SMARCE1 was downregulated in HepG2.2.15 cell compared with the HepG2 cell (Fig. 3c).

### miR-802 promoted HBV infection and replication in the HepG2.2.15 cells

**Fig. 4:**
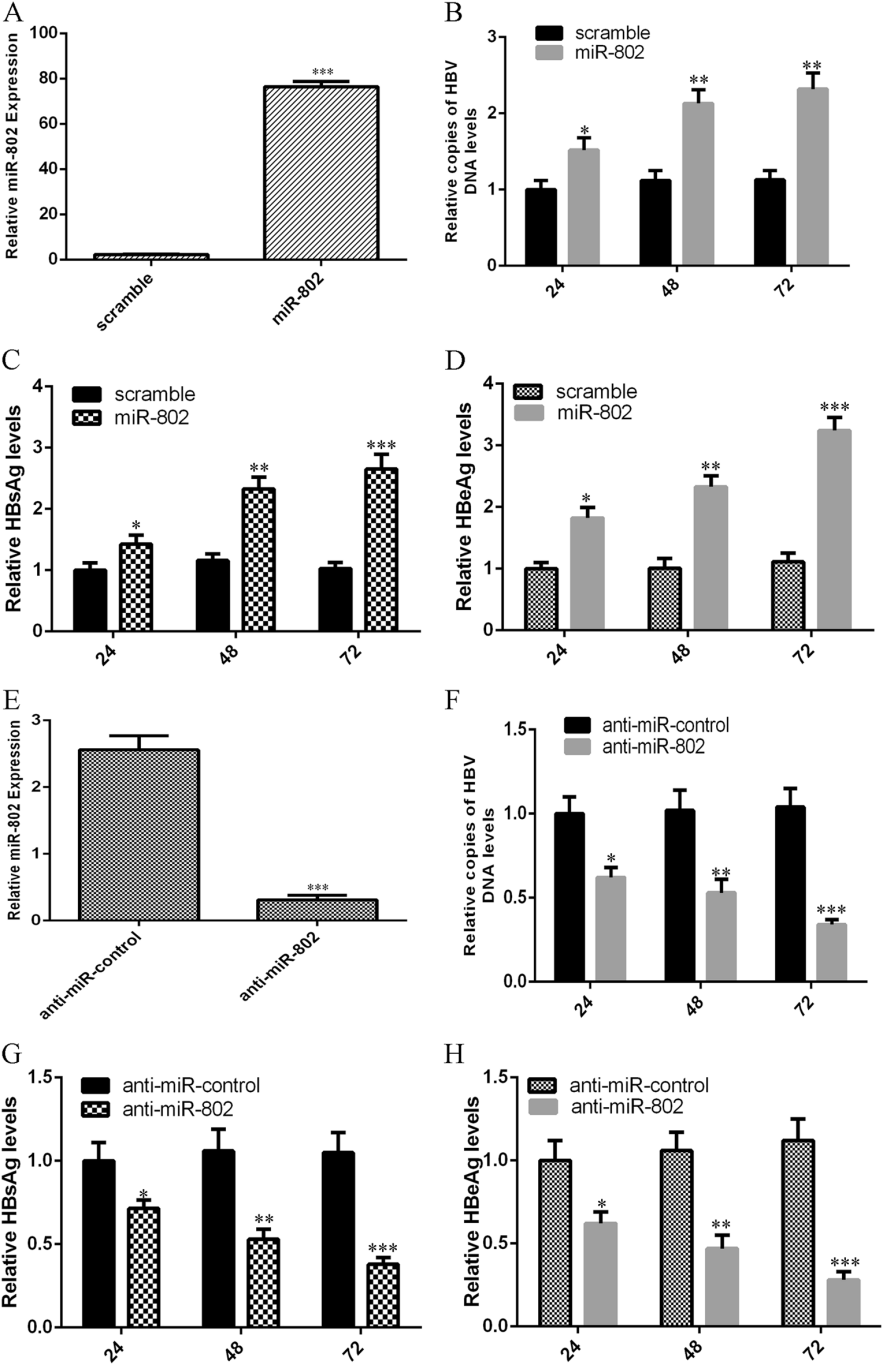
miR-802 promoted HBV infection and replication in the HepG2.2.15 cells. **a** The expression of miR-802 in the HepG2.2.15 cell after transfection with the miR-802 mimic was detected by using qRT-PCR analysis. **b** Ectopic expression of miR-802 significantly enhanced HBV DNA replication. **c** Overexpression of miR-802 promoted the expression of HbsAg in the HepG2.2.15 cell. **d** Ectopic expression of miR-802 increased the HbeAg expression in the HepG2.2.15 cell. **e** The expression of miR-802 in the HepG2.2.15 cell after transfection with anti-miR-802 was assessed by using qRT-PCR analysis. **f** Knockdown of miR-802 significantly decreased the HBV DNA replication. **g** Inhibited expression of miR-802 suppressed the expression of HbsAg in the HepG2.2.15 cell. **h** Knockdown of miR-802 decreased the HbeAg expression in the HepG2.2.15 cell. *p < 0.05, **p < 0.01, and ***p < 0.001. These cells were detected at 24, 48, and 72 h after treatment

To study whether miR-802 affected HBV expression and replication, HepG2.2.15 cell was transfected with miR-802 mimic, scramble, anti-miR-control, and anti-miR-802, respectively. The expression level of miR-802 was significantly upregulated in the HepG2.2.15 cell after treatment with miR-802 mimic (Fig. 4a), and the miR-802 expression was significantly downregulated in the HepG2.2.15 cell after treatment with anti-miR-802 (Fig. 4e). The effect of miR-802 on HBV DNA replication was determined by qRT-PCR, while the expression of HbeAg and HbsAg was measured by ELISA. Our data suggested that ectopic expression of miR-802 significantly enhanced HBV DNA replication (Fig. 4b), while knockdown of miR-802 significantly decreased HBV DNA replication (Fig. 4f). In addition, we showed that overexpression of miR-802 promoted HbsAg (Fig. 4c) and HbeAg (Fig. 4d) expression, while inhibited expression of miR-802 decreased the expression level of HbsAg (Fig. 4g) and HbeAg (Fig. 4h).

### SMARCE1 acted an inhibitory effect on HBV gene replication and expression

**Fig. 5:**
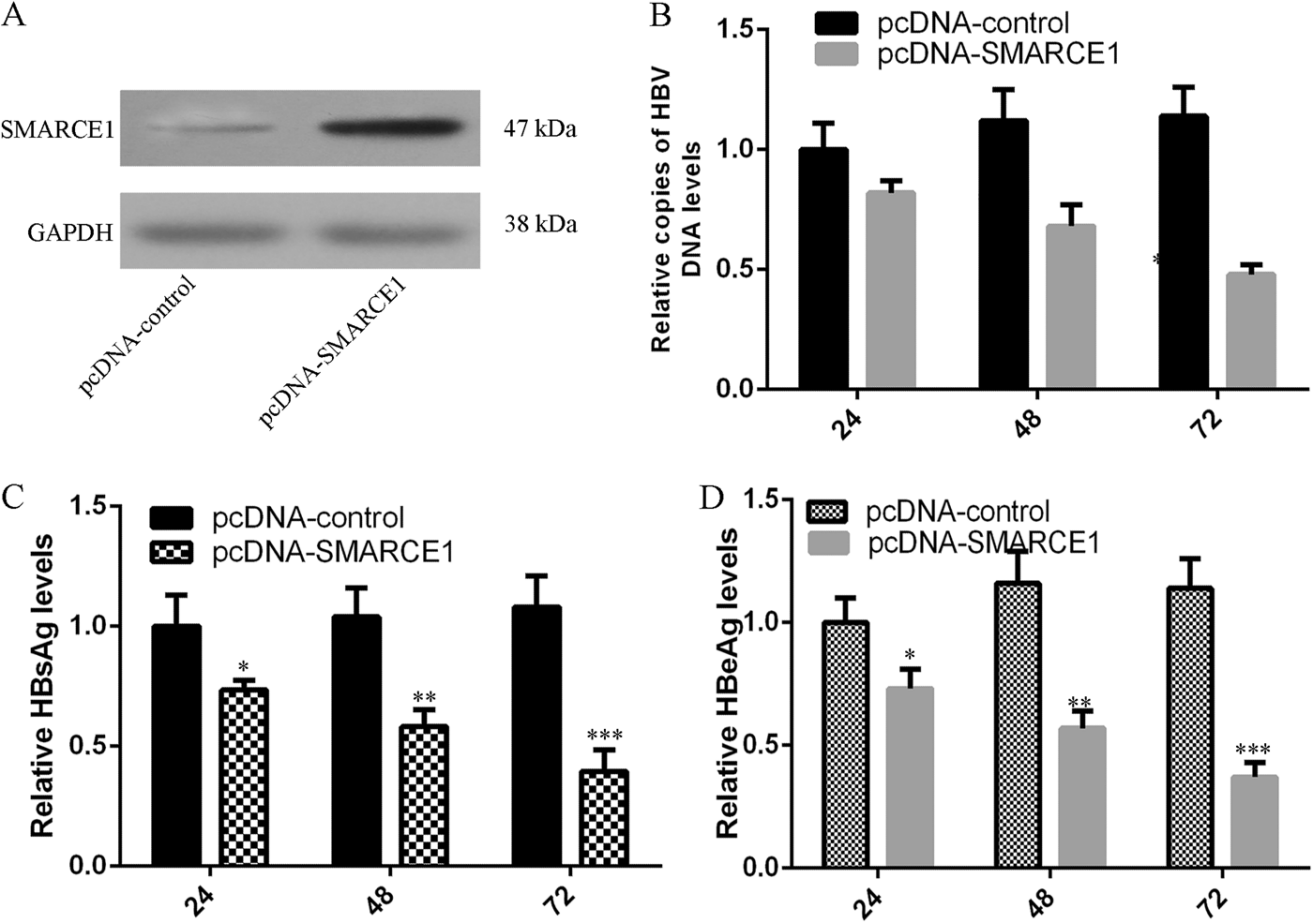
SMARCE1 acted an inhibitory effect on HBV gene replication and expression. **a** The protein expression of SMARCE1 was determined by using western blot. GAPDH was used as the internal control. **b** Ectopic expression of SMARCE1 suppressed HBV DNA replication by using qRT-PCR. **c** Overexpression of SMARCE1 suppressed the HbsAg expression. **d** Elevated expression of SMARCE1 inhibited the expression of HbeAg. *p < 0.05, **p < 0.01, and ***p < 0.001. These cells were detected at 24, 48, and 72 h after treatment

To further study the influence of SMARCE1 on HBV expression and replication, HepG2.2.15 cell was transfected with pcDNA-SMARCE1, pcDNA-control, respectively. We showed that the expression level of SMARCE1 was upregulated in the HepG2.2.15 cell after transfection with pcDNA-SMARCE1 (Fig. 5a). Ectopic expression of SMARCE1 suppressed HBV DNA replication by using qRT-PCR (Fig. 5b). Overexpression of SMARCE1 decreased the expression level of HbsAg (Fig. 5c) and HbeAg (Fig. 5d) by using ELISA assay.

### miR-802 targeted SMARCE1 expression in the HepG2.2.15 cells

**Fig. 6:**
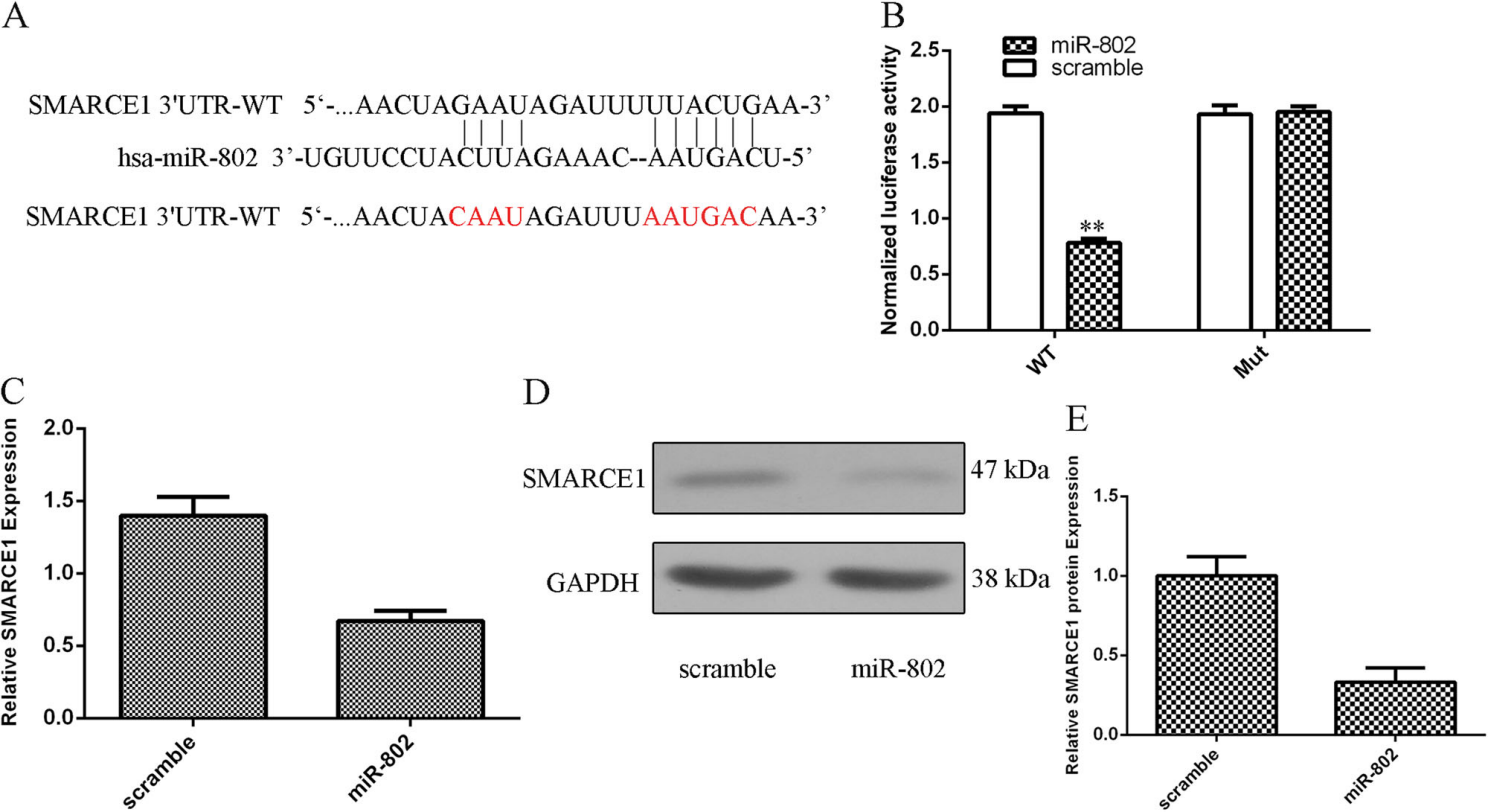
miR-802 targeted SMARCE1 expression in the HepG2.2.15 cells. **a** There were binding sequences between 3′-UTR of SMARCE1 and miR-802 by using miRNA target forecast tool TargetScan. **b** Overexpression of miR-802 suppressed the luciferase activity of WT (wild-type) reporter gene but not MUT (mutant) reporter gene. **c** Ectopic expression of miR-802 suppressed SMARCE1 expression in the HepG2.2.15 cell. **d** The protein expression of SMARCE1 was determined by using western blot. **e** The relative protein signal was shown. **p < 0.01

miRNA target forecast tool TargetScan was exploited to analyze the potential gene target of miR-802. As shown in Fig. 6a, there were binding sequences between 3′-UTR of SMARCE1 and miR-802. In addition, we showed that overexpression of miR-802 suppressed the luciferase activity of WT (wild-type) reporter gene, but not MUT (mutant) reporter gene (Fig. 6b). Moreover, ectopic expression of miR-802 suppressed SMARCE1 expression in the HepG2.2.15 cell (Fig. 6c). Moreover, the protein expression of SMARCE1 was also decreased in the HepG2.2.15 cell after treatment with the miR-802 mimic (Fig. 6d, e).

### miR-802 induced HBV expression and replication through regulating SMARCE1 expression

**Fig. 7:**
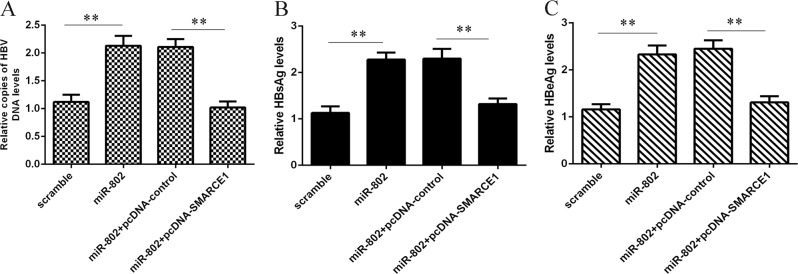
miR-802 induced HBV expression and replication through regulating SMARCE1 expression. **a** miR-802 overexpression promoted HBV replication, whereas co-transfection of pcDNA-SMARCE1 decreased the function of miR-802 on the HepG2.2.15 cells. **b** Ectopic expression of miR-802 promoted the expression of HBsAg, whereas co-transfection of pcDNA-SMARCE1 restored the function of miR-802 on HBsAg expression in the HepG2.2.15 cells. **c** Elevated expression of miR-802 enhanced the expression of HBsAg, whereas co-transfection of pcDNA-SMARCE1 restored the function of miR-802 on HBsAg expression in the HepG2.2.15 cells. **p < 0.01

To investigate whether miR-802 induced HBV expression and replication by modulating SMARCE1, HepG2.2.15 cell was treated with miR-802 scramble or mimics and co-transfected miR-802 with pcDNA-SMARCE1 or pcDNA-control. Our data suggested that miR-802 overexpression promoted HBV replication, whereas co-transfection of pcDNA-SMARCE1 decreased the function of miR-802 on the HepG2.2.15 cells (Fig. 7a). As shown in Fig. 7b, c, ectopic expression of miR-802 promoted the expression of HBsAg and HbeAg, whereas co-transfection of pcDNA-SMARCE1 restored the function of miR-802 on HbeAg and HbsAg expression in the HepG2.2.15 cells.

## Discussion

To ameliorate the therapy of HBV infection, novel therapeutic strategies must be appraised and developed. In our study, we indicated that miR-802 expression was upregulated in the HBV-associated HCC tissues compared with the adjacent noncancerous samples. In addition, we showed that SMARCE1 expression level was downregulated in the HBV-associated HCC tissues compared with the adjacent noncancerous samples. miR-802 expression was negatively related with the expression of SMARCE1 in HBV-associated HCC tissues. Moreover, miR-802 expression was upregulated, and SMARCE1 expression was downregulated in the HBV-infected HepG2.2.15 cells. Ectopic expression of miR-802 significantly enhanced HBV DNA replication, while knockdown of miR-802 significantly decreased HBV DNA replication. We showed that overexpression of miR-802 promoted HbsAg and HbeAg expression, while inhibition of miR-802 decreased the expression level of HbsAg and HbeAg. Furthermore, we indicated that ectopic expression of SMARCE1 suppressed HBV DNA replication and decreased the expression level of HbsAg and HbeAg. Finally, we showed that overexpression of miR-802 promoted HBV DNA replication and HbsAg and HbeAg expression through regulating SMARCE1 expression. These results suggested the important roles of miR-802 on HBV expression and replication, which may offer new insights into the development of potential treatment targets for HBV.

Previous studies indicated that miR-802 played a crucial role in the development of a large number of tumors. For example, Wu et al.^31^ showed that the expression of miR-802 was downregulated in tongue squamous cell carcinoma (TSCC) cell lines and tissues. Overexpression of miR-802 decreased TSCC cell invasion and proliferation through regulating mitogen-activated protein kinase 4 (MAPK4) expression. Yuan et al.^32^ indicated that miR-802 expression was downregulated in breast tumor cells and tissues, and overexpression of miR-802 suppressed breast cancer cell proliferation and tumor growth partly through modulating Forkhead box protein M1 (FoxM1) expression. However, Cao et al.^33^ demonstrated that the expression level of miR-802 was overexpressed in osteosarcoma samples compared with the adjacent normal samples. Ectopic expression of miR-802 induced osteosarcoma cell proliferation partly through regulating p27 expression. Recently, reports on miRNAs have supplied new insights on the potential treatment of HBV infection. Growing number of studies have indicated that the miRNA played crucial roles in HBV expression and replication. However, the role of miR-802 on HBV infection and replication remains unknown. In this study, we proved that miR-802 expression was upregulated in the HBV-associated HCC tissues compared with the adjacent noncancerous samples. Ectopic expression of miR-802 significantly enhanced HBV DNA replication and promoted HbsAg and HbeAg expression.

SMARCE1 is a member of the SNF/SWI family group of the chromatin remodeling complexes, playing a critical role in the regulation of transcription^34^. Previous studies demonstrated that elevated expression of SMARCE1 suppressed HBV expression and replication, while knockdown of SMARCE1 promoted HBV expression and replication^35^. SMARCE1 decreased HBV expression and replication via binding to mutant core promoter of HBV in the HepG2 cells. However, the mechanism of SMARCE1 in the expression and replication of HBV remains largely unknown. By using miRNA target forecast tool TargetScan, there are binding sequences between 3′-UTR of SMARCE1 and miR-802. We indicated that elevated expression of miR-802 inhibited the luciferase activity of WT (wild-type) reporter gene and suppressed SMARCE1 expression in HepG2.2.15 cell. In addition, we showed that SMARCE1 was downregulated in the HBV-infected HepG2.2.15 cells. In line with previous studies, we showed that elevated expression of SMARCE1 inhibited HBV DNA replication and HbsAg and HbeAg expression. Finally, we proved that ectopic expression of miR-802 induced HBV DNA replication and HbsAg and HbeAg expression via regulating SMARCE1 expression. These results indicated that the ability of miR-802 to inhibit SMARCE1 may offer one possible mechanism of post-transcriptional regulation of SMARCE1 in the expression and replication of HBV.

Our research was limited with lack of in vivo experiment, which might weaken the evidence of HBV promotion by miR-802. The narrow species and the lack of the number of tissues and reliable animals susceptible to the HBV infection have limited the study.

In summary, we showed that miR-802 expression was upregulated in the HBV-associated HCC tissues and HBV-infected HepG2.2.15 cells. Enforced expression of miR-802 promoted HBV DNA replication and HbsAg and HbeAg expression through regulating SMARCE1 expression. These data suggested the crucial roles of miR-802 on HBV expression and replication, which might shed new light on the development of novel treatment targets for HBV.
